# 

**Published:** 1891-04

**Authors:** 


TO CONTRIBUTORS.

Articles for publication must reach this office not later than 15th inst.; and are expected to be published exclusively in this JOURNAL. Extra copies of JOURNAL furnished contributors when requested. Reprints by special arrangement. Publication of an article does not necessarily imply endorsement of the views therein expressed.

MEDICAL AND SURGICAL JOURNAL, Post-office box 431.

OFFICE OF SECRETARY
MEDICAL ASSOCIATION OF GEORGIA,
MACON, GA., March 12, 1891.

*Editors Atlanta Medical and Surgical Journal:*

Please allow me to announce in your JOURNAL that the Medical Association of Georgia will hold its forty-second annual session in the city of Augusta, April 15th to 17th, inclusive.

A letter just received from the chairman of the Programme Committee assures me that there will be an attractive and interesting programme arranged for the occasion. The fact that the same committee who gave us such an interesting programme at Brunswick last year is engaged to arrange the programme for this year is a sufficient guarantee that we will have a pleasant and profitable meeting.

Dr. Foster, Chairman of the Programme Committee, writes me that the profession of Augusta have their arms and hearts wide open to give the Association a cordial welcome, and that the city will entertain her guests handsomely; that full and ample hotel accommodations can be had, and that they are very anxious to have a full attendance.

Reduced railroad rates have been secured on all the roads in Georgia upon the certificate plan. Be sure to obtain from the agent where the going ticket is purchased a certificate of such purchase to attend the meeting; and this certificate, when signed by the secretary of the meeting, will entitle the holder to return on one-third the usual fare. If ticket is bought at an intermediate station, at which through tickets to Augusta cannot be purchased, buy to Atlanta, Macon, Albany, Savannah, Athens or Americus, as may be most convenient, and repurchase to Augusta, taking certificates from both agents from whom tickets are secured. The certificates obtained at the above named points will be honored at Augusta, returning to the point at which it was secured; and the other certificate will be honored from thence to the starting point. These tickets good until the 20th, inclusive.

It is very desirable to make this the largest and most profitable session in the history of the Association, and the Association extends a cordial invitation to every graduate of a regular medical college in good standing, to attend the approaching meeting and become a member.

Respectfully and fraternally,
K. P. MOORE, Sec'y.

